# Spectral Similarity Score (SSS)-Barcoding for the Quality Control of LACTEM Emulsifiers by High-Performance Thin-Layer Chromatography

**DOI:** 10.1021/acs.jafc.5c11928

**Published:** 2026-02-26

**Authors:** Katharina Schuster, Sedef Torun, Inès Kainz, Max Schwarz-Blankart, Jörg Hinrichs, Panagiotis Steliopoulos, Claudia Oellig

**Affiliations:** † Department of Food Chemistry and Analytical Chemistry (170a), Institute of Food Chemistry, 26558 University of Hohenheim, Garbenstrasse 28, Stuttgart 70599, Germany; ‡ Department of Soft Matter Science and Dairy Technology (150e), Institute of Food Science and Biotechnology, University of Hohenheim, Garbenstrasse 21, Stuttgart 70599, Germany; § Chemisches und Veterinäruntersuchungsamt (CVUA), Weißenburgerstrasse 3, Karlsruhe 76187, Germany; ∥ Institute of Food Chemistry and Food Biotechnology, 9175 Justus Liebig University Giessen, Heinrich-Buff-Ring 17-19, Giessen 35392, Germany

**Keywords:** high-performance thin-layer chromatography, LACTEM emulsifier, spectral similarity, fingerprint, aerosol whipping cream

## Abstract

LACTEM emulsifiers are widely applied in the food industry to adjust and improve techno-functional properties of food products. The study introduces a high-performance thin-layer chromatography–fluorescence detection (HPTLC–FLD) fingerprint method for the similarity assessment of these emulsifiers using a straightforward barcoding approach based on the concept of spectral similarity scores (SSS), referred to as SSS-barcoding. Analysis of 21 LACTEM emulsifiers showed similarities between two emulsifiers as low as 67%, despite the same product labeling. The method also revealed batch-to-batch variability. Limitations were identified when applying the method to fatty matrices. Finally, partial least-squares regression (PLSR) was applied as a proof-of-concept to predict the techno-functional properties of aerosol whipping cream, such as drainage, apparent viscosity, foam firmness, particle size (D_90,3_), and overrun, from the densitometric data.

## Graphical abstract

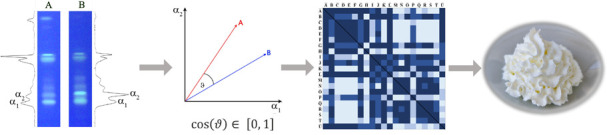

## 1 Introduction

Many food products are complex, multiphased systems.^1^ Surface-active emulsifiers are added to ensure uniform and consistent quality and shelf life stability.^2^ Lactic acid esters of mono- and diacylglycerols (LACTEM), for example, adjust viscosity and improve emulsion and foam stability in aerosol whipping cream. For the structurally related group of mono- and diacylglycerol (MG and DG) emulsifiers, the fatty acid moiety, namely the degree of saturation, strongly influences the techno-functional properties of (aerosol) whipping cream.^3–5^ A recent study also highlighted the role of the polar headgroup of different stearic acid-based emulsifiers in fat crystallization and stabilization of whipping cream.^4^ These findings are consistent with the well-known hydrophilic–lipophilic balance (HLB) concept.^6^ Due to the α-hydroxy structure of lactic acid, esterification with acylglycerols can occur at multiple positions while maintaining reactive hydroxy functions, which can esterify further. In the literature, MG esterified with up to seven^7^ and DG esterified with up to four^8^ lactic acid molecules, respectively, have been reported (Figure 1). However, the structural variability is not reflected in the labeling of LACTEM emulsifiers, even though it may influence the techno-functional properties of food products. This emphasizes the need for studies focusing on the effect of the degree of esterification with lactic acid on techno-functional properties and for reliable analytical methods to characterize LACTEM emulsifiers. Such studies remain scarce, as no defined single reference standards and only mixtures of MG and DG with varying degree of lactylation are commercially available.

**Figure 1:**
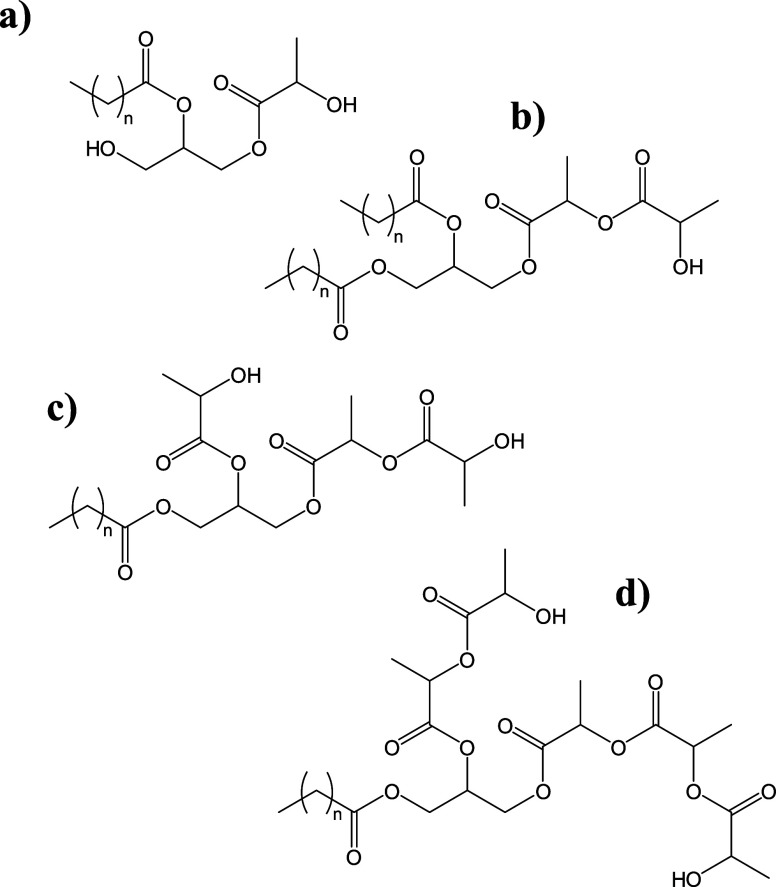
Molecular structures of some LACTEM emulsifier representatives. With: (a) monolactylated 2-MG, (b) dilactylated 1,2-DG, (c) trilactylated 1-MG, (d) pentalactylated 1-MG.

Methods for the qualitative^7–9,11^ and quantitative^10^ analysis of these mixtures in their native form have been described in the literature. In particular, high-performance thin-layer chromatography (HPTLC) has proven to be a reliable tool for the analysis of LACTEM emulsifiers.^8–10^ However, no methods have been described for the quantification of individual emulsifier components. Moreover, the integration of qualitative analysis into quality control through fingerprint analysis has not yet been established.

The comparison of chromatographic fingerprints to assess sample similarity is an emerging approach for quality control of herbal medicines, food, and related products.^12–18^ The similarity of chromatographic fingerprints can be assessed using different approaches, such as unsupervised and supervised methods, as well as similarity parameters. In contrast to unsupervised and supervised methods, which are computationally intensive, similarity parameters such as the correlation coefficient are relatively straightforward to compute. In the literature, this mainly involves the correlation coefficient, which is defined as the cosine of the angle between two centered vectors.^14–18^ This approach using uncentered vectors is also applied in mass spectral library searching, known as spectral similarity,^19^ which is formally equivalent to the congruence coefficient.^14^ High-performance liquid chromatography (HPLC) and gas chromatography (GC) are the chromatographic techniques most commonly applied in similarity assessment, whereas HPTLC is used only in specialized applications. HPTLC offers specific features such as parallel analysis of multiple samples, short analysis times, and relatively low running costs. Automation and possible hyphenation to various detection methods, such as ultraviolet (UV) detection, fluorescence detection (FLD), and mass spectrometry (MS), make it a versatile chromatographic technique that complements HPLC and GC. This qualifies HPTLC as well-suited for characterizing LACTEM emulsifiers based on their chromatographic fingerprint. Despite the potential of spectral similarity and the calculated spectral similarity scores (SSS) for chromatographic fingerprint comparison, their application for densitometric data obtained through HPTLC for the analysis of food emulsifiers remains unexplored. Thus, this study extends spectral similarity from mass spectrometry to densitometric HPTLC fingerprints of LACTEM emulsifiers and assesses compositional changes in emulsifier batches and LACTEM-containing products. Additionally, the potential to predict the techno-functional properties, such as drainage, apparent viscosity, foam firmness, particle size (D_90,3_), and overrun of (aerosol) whipping cream, from the densitometric data using partial least-squares regression (PLSR) was evaluated.

## 2 Materials and Methods

### 2.1 Chemicals

Acetone (Rotisolv, Pestilyse, ≥99.8%) and methylene chloride (Rotisolv, HPLC, ≥99.9%) were purchased from Carl Roth (Karlsruhe, Germany). Chloroform (Chromasolv, for residue analysis, ≥99.9%) and methyl *tert*-butyl ether (MTBE, Chromasolv, for HPLC, ≥99.8%) were purchased from Honeywell Riedel-de Haën (Charlotte, NC, US). Diethyl ether (≥99.5%) was from Thermo Fisher Scientific (Waltham, MS, US). Ethanol (absolute for HPLC, ≥99.9%), *n*-heptane (Chemsolute, for HPLC, ≥99.2%), and methanol (Chemsolute, LC–MS, >99.9%) were obtained from Th. Geyer (Renningen, Germany). Ammonium formate (LC–MS grade) and hydrochloric acid (37%) were from Fisher Scientific (Hampton, NH, US). Boric acid (>99.8%), ethylene glycol (≥99%), potassium carbonate (K_2_CO_3_, anhydrous, for analysis, ≥99%), pyridine (≥99%), and sodium hydrogen carbonate (≥99%) were purchased from Carl Roth. Formic acid (≥99%) was from VWR Chemicals, 2-naphthoyl chloride (>98%) from TCI (Tokyo, Japan), and primuline (50% dye content) from Sigma-Aldrich (Burlington, MA, US). Magnesium chloride (anhydrous, for synthesis) and HPTLC silica gel 60 F_254_ MS-grade glass plates (20 × 10 cm) were obtained from Merck (Darmstadt, Germany).

### 2.2 Samples and Sample Solutions

A set of 21 LACTEM emulsifiers (A-U), eight LACTEM-containing products (AA-AH, composition Table S1), three ACETEM (acetic acid esters of MG/DG), three CITREM (citric acid esters of MG/DG), two DATEM (diacetyl tartaric acid esters of MG/DG), and two MG/DG emulsifiers were obtained from emulsifier and food companies, and were stored at 4 °C prior to analysis. For HPTLC–FLD analysis, sample solutions with a concentration of 500 ng μL^–1^ were prepared in MTBE and stored in the refrigerator prior to and between analyses. An internal standard (IS, Section 2.3) was added, resulting in a concentration of 18 ng μL^–1^. For the LACTEM-containing products (AA-AH), the CITREM and the DATEM samples, the sample solution included heating the samples in an airtight bottle at 80 °C for 5 min.

### 2.3 Preparation of the Internal Standard (IS)

The IS (1,2-bis naphthoylethanediol) was synthesized according to Schuster et al. (2023) with slight modifications.^10^ The organic phase containing the synthesis product was evaporated to dryness and reconstituted in MTBE. After centrifugation, the supernatant was evaporated to dryness again, the mass of the residue was determined gravimetrically, and the IS was dissolved in MTBE and diluted to a concentration of 1800 ng μL^–1^.

### 2.4 Manufacturing of Aerosol Whipping Cream and Determination of Techno-Functional Properties

Aerosol whipping cream with an emulsifier content of 0.8 g 100 g^–1^ was manufactured according to Schuster et al. (2025).^9^ Techno-functional properties, namely particle size (D_90,3_), apparent viscosity, overrun, foam firmness, and normalized drainage, were analyzed according to Schuster et al. (2025) with three replicates, each with two repetitions.^9^

### 2.5 High-Performance Thin-Layer Chromatography–Fluorescence Detection (HPTLC–FLD)

HPTLC–FLD analysis was performed according to Schuster et al. (2023) with minor variations that are described in the following section.^10^ 20 cm × 10 cm HPTLC silica gel 60 F_254_ MS-grade plates were prewashed with methanol, followed by drying of the plates in a drying cabinet at 100 °C for 30 min. Next, plates were impregnated with a boric acid solution containing 1% boric acid in ethanol/water (4:1, *V/V*) using a TLC immersion device II (time: 2, speed: 1, CAMAG, Muttenz, Switzerland). After dipping, plates were briefly dried under a stream of cold air and subsequently placed on a plate heater (CAMAG) at 100 °C for 1 h. After cooling at room temperature, the plates were stored in a SICCO star vitrum desiccator (Bohlender, Grünsfeld, Germany) prior to analysis. Ten μL of sample solution (resulting in 5 μg of sample per zone) were applied bandwise (band length of 5 mm) on the plate with an Automatic TLC Sampler 4 (ATS4, CAMAG). 2-fold development was performed in an Automatic Developing Chamber (ADC2, CAMAG). Before both developments, a saturated magnesium chloride solution controlled the plate activity to 33% relative humidity. Prior to the first development, the chamber was saturated with the mobile phase for 15 min using filter paper. For test purposes, the original mobile phase compositions by Schuster et al. (2023) were used for the first (chloroform/methanol/water/formic acid (6.7:0.6:0.12:0.02, *V/V*) up to 50 mm) and second (*n*-heptane/diethyl ether/formic acid (5.5:4.5:0.1, *V/V*) up to 80 mm) development.^10^ Final adjusted mobile phase compositions for both developments were as follows: chloroform/methanol/water (8:0.8:0.12, *V/V*) up to a migration distance of 50 mm, followed by a 10 min drying step, and *n*-heptane/diethyl ether (5.5:4.5, *V/V*) up to 80 mm, followed by a 5 min drying step. After dipping in primuline (0.05% in acetone/water (4:1, *V/V*)), the plates were stored for 1 h in a desiccator at 47% relative humidity adjusted with saturated K_2_CO_3_ solution. Plate images were captured after every working step at UV 254 nm and UV 366 nm. Densitograms were obtained at UV 254 nm in absorption mode prior to dipping in primuline, and UV 366 nm in fluorescence mode (optical filter of K400 and an analog offset of 10%) using the TLC Scanner (CAMAG). The HPTLC instruments were controlled with the software winCATS, version 1.4.6.2002 (CAMAG).

### 2.6 Data Analysis and Statistics

Robustness was evaluated by assessing the variability of chromatographic separation, as indicated by a low coefficient of variation (*CV*) for *h*R_F_ values, the retardation factor, which is the ratio of the migration distance of a substance to the distance of the application position to the solvent front, multiplied by 100. The *CV* of the *h*R_F_ values was calculated according to (1), where *s* is defined as the standard deviation, and *x̅* as the mean.

1 $$CV\;\left(\%\right)=\frac{s}{\mathrm{x̅}}\cdot 100$$

To detect significant differences in *h*R_F_ values between two groups, a two-sided student’s *t*-test was performed at a significance level of 0.05. Testing for significant differences in *s* was performed using the F-test at a significance level of 0.05. To detect significant differences in *h*R_F_ values between three or more groups, the assumptions of homogeneity and normality were assessed using Levene’s test (*p* > 0.05) and Shapiro–Wilk test, respectively (*p* > 0.05). If both assumptions were met, one-way analysis of variance (ANOVA) was performed. If one or both assumptions were not met, Welch’s ANOVA or Kruskal–Wallis test was applied. Calculation of the *CV*, student’s *t*-test, and F-test were performed using Microsoft Excel (Version 2505, Microsoft Corporation, Redmond, WA, US). Levene’s test, Shapiro–Wilk test, ANOVA, Welch’s ANOVA, and Kruskal–Wallis test were performed using RStudio, version 2024.09.0 (Posit Software, PBC, Boston, MA, US) running on R, version 4.3.1 (R Foundation for Statistical Computing, Vienna, Austria).

### 2.7 Data Pre-Treatment

The data pretreatment approach involved smoothing of the raw data (Savitzky–Golay 7) and baseline correction (lowest slope), both performed using winCATS (CAMAG). This was followed by the export of densitometric data acquired at UV 366 nm (fluorescence mode) and UV 254 nm (absorption mode) as a .csv file. Maximum signal intensities of eight common peaks (α_1–4_, β_1,2_, γ_1_, δ_1_) characteristic of LACTEM emulsifiers (UV 366 nm) and the IS (UV 254 nm) were extracted semiautomatically in predefined *h*R_F_ value intervals using Microsoft Excel (Version 2505; Microsoft Corporation, Redmond). The intensities of the common peaks were then normalized to the signal intensity of the IS for each sample.

### 2.8 Spectral Similarity Scores (SSS)

#### 2.8.1 Calculation of Spectral Similarity Scores (SSS)

SSS of the 21 LACTEM emulsifiers were computed using the cosine angle method. Mathematically, the spectral similarity cos θ of two densitograms represented as vectors A⃗ and B⃗ is calculated by dividing the dot product of the two vectors by the product of their lengths (2), as follows

2 $$\cos\theta =\frac{\sum_{i=1}^{n}{\mathrm{A⃗}}_{i}{\mathrm{B⃗}}_{i}}{\sqrt{\sum_{i=1}^{n}{\mathrm{A⃗}}_{i}^{2}}\sqrt{\sum_{i=1}^{n}{\mathrm{B⃗}}_{i}^{2}}}$$

To compute the SSS from the pretreated data (Section 2.7), Microsoft Excel (Version 2505, Microsoft Corporation) was used. For the generation of reference fingerprints and thresholds for authentic samples, the 21 LACTEM emulsifiers were analyzed in 6 to 8 replicates by HPTLC–FLD (Section 2.5). The relative intensities of each signal were averaged for each emulsifier, and the resulting mean values were used as the references for the calculation of the SSS of all subsequent measurements.

#### 2.8.2 Assessment of Spectral Similarity Score (SSS) Variability

To evaluate interplate deviation (*n* = 6–8), the ratios of signal α_1_ relative to each peak were calculated using normalized signals, and the relative half-widths of the 95% confidence interval (*U*) were calculated. Ratios were classified based on their *U* values (*U* ≤ 40% or *U* > 40%). For the analysis of SSS, calculations were performed using both the complete set of signals and a reduced set excluding α_4_ and γ_1_. SSS were calculated for both sets for each plate, and emulsifier against the reference fingerprint (average intensities of each peak), and the ΔSSS were calculated by subtracting the SSS_reduced_set_ from the SSS_full_set_.

### 2.9 Application of SSS-Barcoding

Eight LACTEM-containing products (AA-AH) and ten non-LACTEM food emulsifiers (three ACETEM, three CITREM, two DATEM, two MG/DG) were analyzed (*n* = 4 and 2, respectively) with the optimized HPTLC–FLD method. SSS were calculated relative to the LACTEM references and visualized as SSS-barcode plots. To assess the batch-to-batch variability of LACTEM emulsifiers, two batches of four emulsifiers (N, O, P, Q), obtained from two different years, were analyzed in duplicate with the optimized HPTLC–FLD method. SSS were calculated relative to the LACTEM references. SSS-barcode plots were visually compared, and the differences of the SSS for batches of the same emulsifiers were calculated.

### 2.10 Partial Least Squares Regression (PLSR)

Eight LACTEM emulsifiers (D, O, P, R) and LACTEM-containing products (AB, AC, AE, AH) were selected based on differences in their SSS-barcode pattern. Emulsifiers were analyzed in four replicates by HPTLC–FLD (Section 2.5). Aerosol whipping cream manufacturing and assessment of techno-functional properties were performed according to Section 2.4. Due to the laborious manufacturing and assessment of the techno-functional properties, the sample size was low (four LACTEM and four LACTEM-containing products). Data pretreatment was performed according to Section 2.7. Prior to PLSR, the data was logarithmically transformed to the base of 10. Scaling, which involved standardization of the variables, was not applied. For each techno-functional property, the optimal number of components was determined based on minimum root mean squared error of prediction (RMSEP). Leave-one-out-cross validation (LOO) was used for the validation of the model. In addition, the RMSEP of the residues between the measured variable and the predicted variable was calculated. To check for overfitting, the ratio of the RMSEP_LOO_ and the RMSEP_Residues_ was calculated. A ratio of >1.5 was considered an indicator for overfitting. PLSR was performed using the package pls in RStudio, version 2024.09.0 (Posit Software, PBC) running on R, version 4.3.1 (R Foundation for Statistical Computing).

## 3 Results and Discussion

### 3.1 Terminology

In the literature, the terms profiling and fingerprinting are not clearly defined, leading to inconsistent use.^20–23^ Based on these definitions, we defined our approach as chemical fingerprinting. No quantification or identification of the analytes was performed in the current study; however, quantification is possible, as shown in previous studies.^10^ Formally, a preselection of analytes is performed due to the derivatization step with primuline. However, as per definition, LACTEM emulsifiers are lactic acid esters of MG and DG, and only minor constituents are not visualized with the visualization technique. Furthermore, according to Stilo et al. (2021), a fingerprint can also be recorded from a particular fraction of compounds.^22^ For similarity analysis, common peaks were selected, as the goal of the study was to minimize computational effort. Nevertheless, the whole densitogram is always recorded and can be evaluated to check for unusual peaks.

### 3.2 Optimization of High-Performance Thin-Layer Chromatography–Fluorescence Detection (HPTLC–FLD) Parameters

#### 3.2.1 Definition of Common Peaks and Reduction of Inter-Plate Intensity Variation

Starting from the HPTLC–FLD method by Schuster et al. (2023),^10^ several factors related to the interplate robustness of the method were evaluated. For quantification, samples and calibration standards are applied to the same plate. Thus, interplate differences regarding signal intensity ratios or isomerization are less critical, as LACTEM emulsifiers are quantified as the total amount of MG and lactylated MG.^10^ In contrast, for the similarity analysis of chromatographic fingerprints, achieving comparable results between plates over time and across plates is of crucial importance because a reference fingerprint is typically created and then used as a basis for all subsequent samples. In the literature, this reference fingerprint is generated by calculating the mean signal intensities of so-called common peaks of authentic samples.^15^ These common peaks appear in all authentic samples.^16^ A robust analysis technique is therefore essential to ensure consistent results in similarity analysis.

For all LACTEM emulsifiers, eight characteristic peaks were detected in the densitogram obtained at UV 366 nm, defined as common peaks. For clarity, the peak assignment is illustrated in a densitogram obtained after method optimization (Figure 2). Four signal groups were defined, according to mass spectrometric analysis (data not shown) by Oellig et al. (2020):^8^ α_1–4_: MG and their lactic acid esters, β_1–2_: DG and their lactic acid esters, γ_1_: free fatty acids (FA), and δ_1_: triacylglycerols (TG).

**Figure 2:**
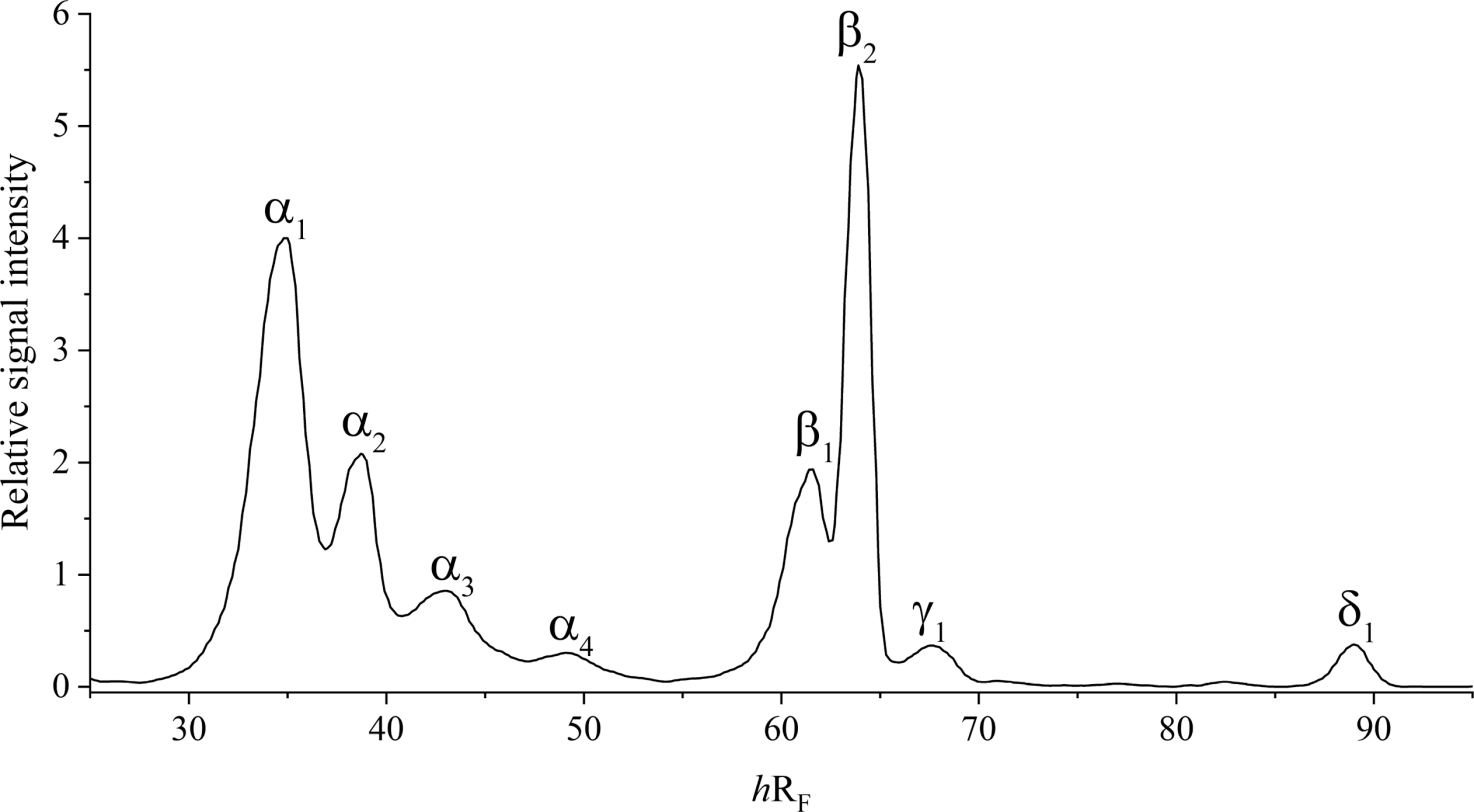
Densitogram of LACTEM emulsifier A obtained at UV 366 nm (optical filter of K400; optimized method) in fluorescence mode. Signal intensities were normalized to the internal standard. With: α_1–4_: monoacylglycerols and their lactic acid esters, β_1–2_: diacylglycerols and their lactic acid esters, γ_1_: free fatty acids, and δ_1_: triacylglycerols. HPTLC plates were developed using solvent mixtures of chloroform/methanol/water (8:0.8:0.12, *V/V*) and *n*-heptane/diethyl ether (5.5:4.5, *V/V*) for the first and second development, respectively.

Applying the original solvent compositions by Schuster et al. (2023), the intensity ratio of the α peaks showed plate-to-plate variability when analyzed under the same conditions (Figure S1), indicating possible isomerization of the LACTEM components. Acyl migration of acylglycerols with free hydroxy groups is a known phenomenon.^24,25^ Further, it has been described that the acidity of silica gel promotes acyl migration for MG in column chromatography.^25^ To prevent acyl migration of acylglycerols, plates can be impregnated with borate or boric acid.^26,27^ Acyl migration is prevented by the interaction of the boric acid with the free hydroxy groups of the acylglycerols.^27^ Additionally, boric acid can improve the separation of isomers.^28^ Impregnation of the plate with 1 and 2% boric acid (ethanol/water (4:1, *V/V*), concentrations were chosen based on internal pre-experiments), decreased the chromatographic separation within the α group (Figure 3 (1) vs (2)). Hence, the mobile phases were adjusted. Omitting formic acid for both developments and increasing the share of chloroform and methanol for the first development resulted in comparable chromatographic separation with the original method. However, the signals of the α group exhibited slightly higher but reproducible *h*R_F_ values. Additionally, alterations in the signal fine structure were observed (Figure 3 (3)). Using 1% boric acid for impregnation prior to the application, interplate changes in signal ratios were no longer observed. Within the scope of the current study, no influence of the boric acid impregnation on the fluorescence behavior was detected. The boric acid solution was freshly prepared every month.

**Figure 3:**
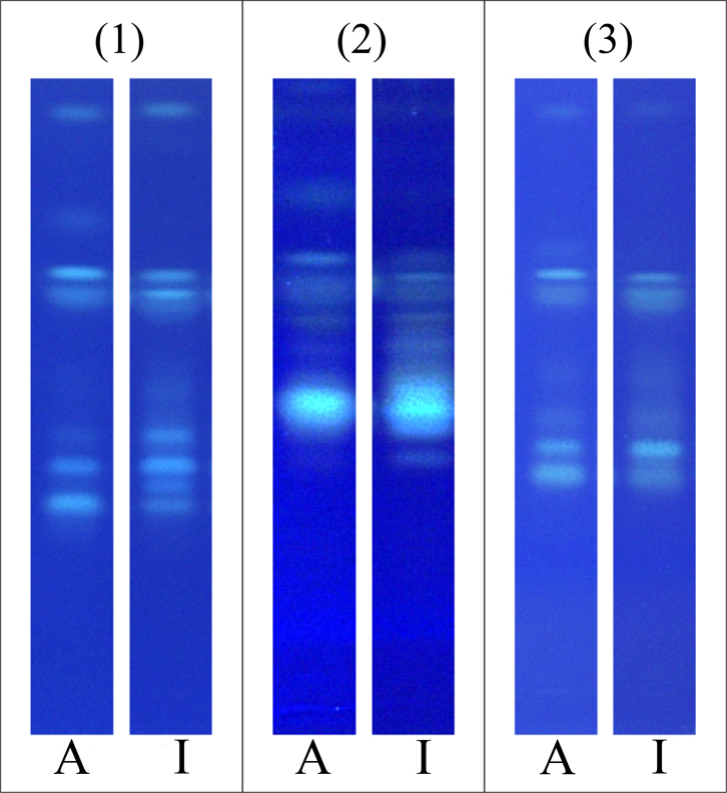
Separation of LACTEM emulsifiers A and I on HPTLC silica gel 60 F_254_ MS-grade plates **(1)** according to Schuster et al. (2023) applying solvent mixtures of chloroform/methanol/water/formic acid (6.7:0.6:0.12:0.02, *V/V*) and *n*-heptane/diethyl ether/formic acid (5.5:4.5:0.1, *V/V*) for first and second development, respectively,^10^ **(2)** with 1% boric acid impregnation prior to sample application, as a modification of (1) applying solvent mixtures of chloroform/methanol/water/formic acid (6.7:0.6:0.12:0.02, *V/V*) and *n*-heptane/diethyl ether/formic acid (5.5:4.5:0.1, *V/V*) for first and second development, respectively,^10^ and **(3)** with boric acid impregnation and adjusted mobile phase consisting of chloroform/methanol/water (8:0.8:0.12, *V/V*) and *n*-heptane/diethyl ether (5.5:4.5, *V/V*) for first and second development, respectively, visualized under UV 366 nm after impregnation with primuline.

#### 3.2.2 Influence of Chamber Saturation and Plate Conditioning on *h*R_F_ Value Variability

Using the optimized mobile phase composition, the variability of the chromatographic separation was determined by analyzing each two plates on 3 days. The determined *CV* varied between the different signal groups, with *CV* between 6.8 and 8.5% for the α group and 0.6 to 2.7% for the β, γ, and δ groups (Figure 4). This might be related to the two different mobile phases required for the analysis of LACTEM emulsifiers. Derivatization of the HPTLC plate after the first development (chloroform/methanol/water, 8:0.8:0.12, *V/V* up to 50 mm) showed that the *h*R_F_ values of the α group were mainly influenced by the first development, where more unpolar compounds, such as TG, DG, and lactic acid esters of DG, migrated in the solvent front. A chromatographic separation of the latter was only achieved in the second development (*n*-heptane/diethyl ether, 5.5:4.5, *V/V* up to 80 mm). It must be noted that the mobile phase for the first development is a two-phase system, which must be shaken well to avoid phase separation. Thus, this was considered a critical factor with a strong influence on the chromatographic separation. Comparing the intraplate variation (Table S2) with the interplate variation (Table S3) of the *h*R_F_ value, it was also shown that the intraplate variation for the α group was considerably lower (0.6 to 1.3%) than the interplate variation (6.8 to 8.5%). This was not the case for the other signals, supporting the hypothesis and underlining the importance of the chamber condition.

**Figure 4:**
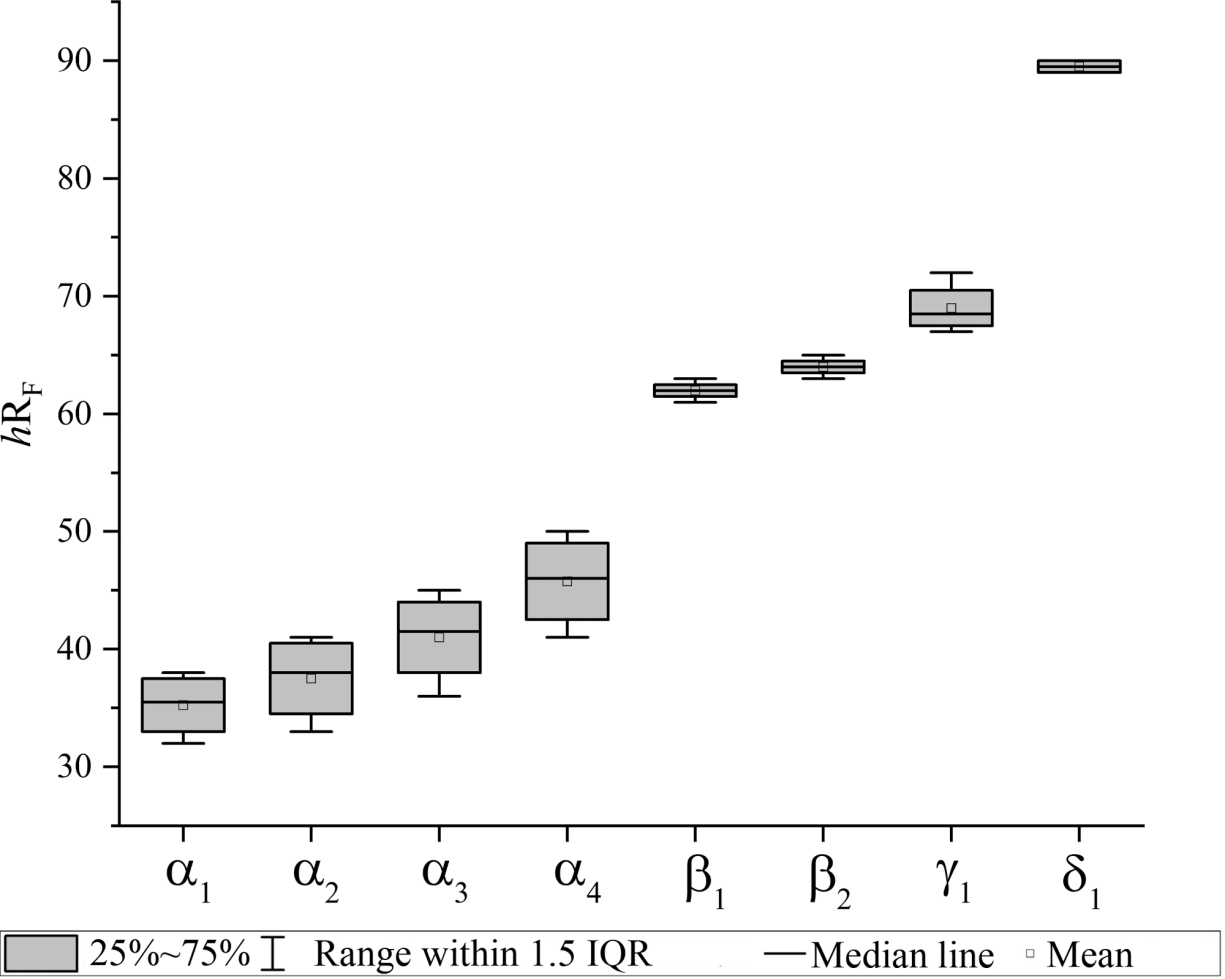
Box plot with 25 and 75% quantiles, the median, the mean, and 1.5 the interquartile range (IQR) of the *h*R_F_ values of the signals α_1_ to δ_1_ of LACTEM emulsifier A (*n* = 4).

To assess the influence of chamber saturation with the mobile phase, four LACTEM emulsifiers were applied as duplicates on boric acid-impregnated plates, and analyzed in quadruplicate by HPTLC–FLD (Section 2.6). Plates were analyzed under four different conditions: (a) no chamber saturation, (b) chamber saturation before the first development, (c) chamber saturation before the second development, and (d) chamber saturation before both developments. Chamber saturation took place automatically in the ADC2. Conditioning of the plate with the mobile phase was performed similarly to chamber saturation. Finally, a setup combined chamber saturation and plate conditioning to assess potential synergistic effects. A decreasing trend for the *CV* for specific signals (α_1_ and α_2_) was observed when chamber saturation or plate conditioning was applied prior to the first development (Table S2). The combination of chamber saturation and plate conditioning resulted in the same variability as observed with chamber saturation alone. Thus, chamber saturation was used prior to the first development in the final method. Applying the F-test, no statistically significant differences (*p* > 0.05) in variance were observed between the method without chamber saturation and plate conditioning and the modified approaches. Nonetheless, given the consistent trend toward reduced variability for multiple signals and the relatively small sample size (*n* = 4), chamber saturation prior to the first development was retained for further experiments. To finally examine the stability of the boric acid impregnation, six plates impregnated with 1% boric acid (Section 2.6) were used for duplicate analysis of the 21 LACTEM emulsifiers by HPTLC–FLD on days 0, 2, and 4, covering one working week. Storage experiments of the boric acid-impregnated plates revealed no significant change in *h*R_F_ values (*p* > 0.05, Table S4) over a one-week period, indicating that the impregnation with respect to the *h*R_F_ values was at least stable for this period.

#### 3.2.3 Influence of Post-Impregnation Desiccator Storage on Signal Intensity

Visualization with primuline is a widely used method for the nonspecific detection of lipids in HPTLC.^29^ Primuline binds noncovalently to the acyl chains of lipids, resulting in an increase in fluorescence at UV 366 nm.^30^ In the current study, the duration of the postimpregnation storage time in the desiccator at a relative humidity of 47% (adjusted with saturated K_2_CO_3_) was identified as a critical parameter affecting signal intensity, and thus, the signal-to-noise ratio. This can be attributed to the water content of the layer and has already been described before.^31^ The (relative) signal intensity increased with increasing storage time in the desiccator postimpregnation, reaching a plateau at approximately 1 h (Figure 5). This observation was made for three plates on three different days. As for the calculation of SSS, only the angle between the two vectors is of importance; the absolute signal intensities do not matter. Instead, signal ratios should be constant. Notably, for different LACTEM compounds, different slopes for the increase in signal intensity were observed (Figure 5, signal α_3_ and γ_1_), highlighting the need for controlled parameters. Based on these findings, a minimum desiccator storage time of 1 h postimpregnation was defined for the final method.

**Figure 5:**
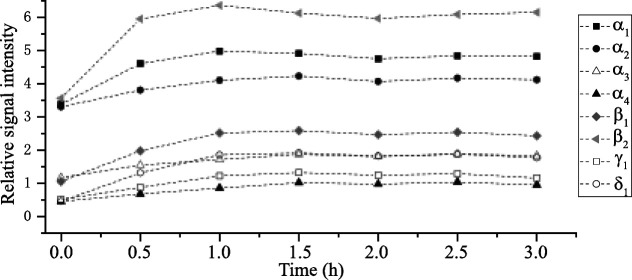
Relative signal intensity (normalized to the internal standard) of common LACTEM emulsifier signals in relation to postprimuline impregnation storage time in the desiccator, exemplarily shown for LACTEM emulsifier A. The plate was scanned at UV 366 nm in fluorescence mode between 0- and 3-h postimpregnation, and stored in the desiccator at a relative humidity of 47% after impregnation.

### 3.3 Spectral Similarity Scores (SSS)

The SSS provides a quantitative measure of similarity between chromatographic or spectroscopic fingerprints based on the cosine similarity. Two densitograms A and B can be represented as vectors A⃗ and B⃗ in an *n*-dimensional space $R^{n}$ . Each dimension corresponds to a discrete *h*R_F_ value, and the components reflect the respective signal intensity at a given *h*R_F_ value. Similarity is quantified by the cosine of the angle θ between the two vectors (Figure 6). The values range from −1 ≤ SSS ≤1. When using only positive signals, the range is restricted to 0 ≤ SSS ≤1, where 0 indicates maximum dissimilarity (orthogonal vectors), and 1 a maximum similarity (congruent vectors, i.e., an angle of 0° between them).

**Figure 6:**
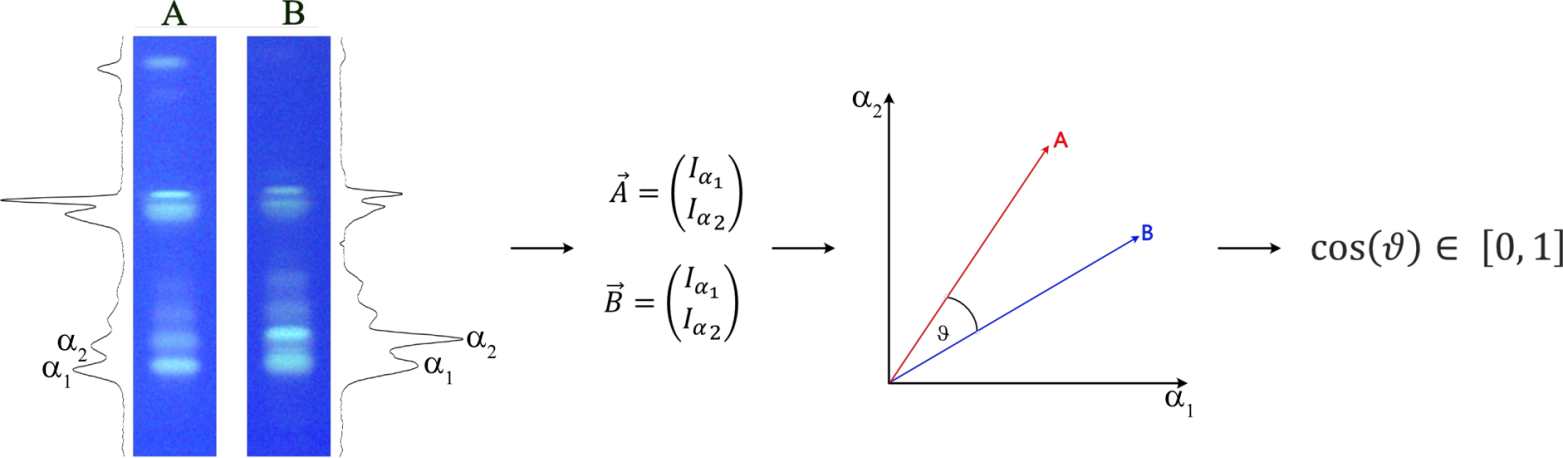
Visualization of the concept of spectral similarity exemplarily shown for two signals (α_1–2_) for two emulsifiers.

In the present study, peak intensities rather than peak areas were used to calculate the SSS because some signals, mainly the α and β group signals, were not baseline-resolved. However, peak intensities can be affected by peak broadening or variations in signal shape. Thus, each densitogram must be evaluated carefully.

#### 3.3.1 Variability of SSS

When analyzing the interplate variability, a significant positive correlation was observed between the relative signal intensity ratio of signal α_1_ to a specific signal and the *U* (Table S5) based on linear regression (*p* < 0.01, *R* ^2^ = 0.41). This indicates that *U* increases with increasing ratio of signal α_1_ to a specific signal (Figure S2), which corresponds to smaller specific signals. Overall, *U* was below the set threshold of 40% for 93% of the signals. Nevertheless, the strongly deviating low-intensity signals with a *U* > 40% (α_4_ and γ_1_) might influence the robustness of the SSS. A comparative analysis of the SSS of the complete set of signals and a reduced set of signals, excluding the lower intense signals α_4_ and γ_1_ correlating with a higher *U*, was conducted. Across all plate–emulsifier combinations, the mean ΔSSS was 0.005, which was considered acceptable. Only two emulsifiers (B and G) showed elevated ΔSSS, which were detected across all plates (Figure S3). Based on these results, the complete set of signals was retained as they are characteristic for LACTEM emulsifiers. Thus, omitting them could lead to a loss of information, and keeping them does not lead to deviating SSS.

#### 3.3.2 Calculating Thresholds for Color-Coding

For visualization of the SSS, establishing thresholds for color-coding is essential to generate a barcode-like identification for each emulsifier. In chemical fingerprinting, a threshold of >0.9 is often considered sufficient for classification purposes.^32^ Other authors use only fingerprints with a correlation coefficient of >0.75 for the generation of the reference fingerprint and establish this value as a threshold.^15^ Additionally, the minimal correlation coefficient is applied in the analysis of authentic samples for threshold setting.^33^ Recently, Alaerts et al. (2012) proposed a method for calculating thresholds based on the calculation of warning and control limits for control charts.^14^ Assuming normal distribution of the data, warning limits were defined as the *r̅*–1.96s_r_, and control limits as the *r̅*–(3.09s_r_), with *r̅* the average correlation coefficient and *s*_*r*_ the standard deviation of the correlation coefficients. For our approach using SSS, we adapted this principle accordingly and calculated thresholds based on the average self-comparison SSS ( $\overset{®}{\mathrm{SSS}}$ = 0.9885, *s*_SSS_ = 0.0171) to the LACTEM references to define similarity categories for visualization. Intervals for color-coding were defined starting with $\overset{®}{\mathrm{SSS}}$ −(1.96·s_SSS_), and then, extending the range stepwise with multiples of 1.96 to $\overset{®}{\mathrm{SSS}}$ −(1.96·2·s_SSS_), and $\overset{®}{\mathrm{SSS}}$ −(1.96·3·s_SSS_), leading to the following color-coding intervals: 1) [1, 0.9519­[, 2) [0.9519, 0.9182­[, 3) [0.9182, 0.8846­[, and 4) [0.8846, 0].

#### 3.3.3 SSS-Barcoding for the Comparison of LACTEM Emulsifiers

Applying the calculated thresholds for color-coding (Section 3.3.2) on the SSS resulted in a correlation matrix plot similar to a heat map. The application of the SSS-barcoding offers two insights: (1) it provides an intuitive visualization of differences between densitograms of emulsifiers, and (2) each segment of the SSS-barcode corresponds to the exact similarity between an emulsifier and the reference.

Calculating the SSS between the references (Table S6) and themselves showed that the investigated LACTEM emulsifiers revealed characteristic SSS barcode plots (Figure 7A). These SSS-barcodes can be clustered into four groups, thus supporting the visually identified groupings made by Schuster et al. (2023)^10^ using quantitative measurements. However, SSS-barcoding did not fully match the visual classification, indicating subtle differences in similarity patterns not apparent by eye. Furthermore, the method at the time was not optimized to get constant peak ratios across all plates. Figure S4 shows the SSS-barcodes sorted by group for visualization purposes. The underlying densitograms of the groups will not be discussed in detail, as they have already been published elsewhere.^10^ The average SSS across all emulsifier combinations was 0.9179. The overall minimum SSS was 0.6658, observed between emulsifier H and reference N, which are categorized into different groups, highlighting compositional differences between LACTEM emulsifiers, although they are declared under the same name, LACTEM. Underlying structural variations might be due to several reasons, such as the esterification degree with lactic acid, which can range from one to seven^7^ and four^8^ or more lactic acid molecules per MG and DG, respectively. Additionally, different ratios of MG, DG, TG, and free FA, as well as fatty acid constitution, contribute to structural variability. In this context, it has already been shown that the degree of saturation influences the fluorescence behavior of primuline.^31^

**Figure 7:**
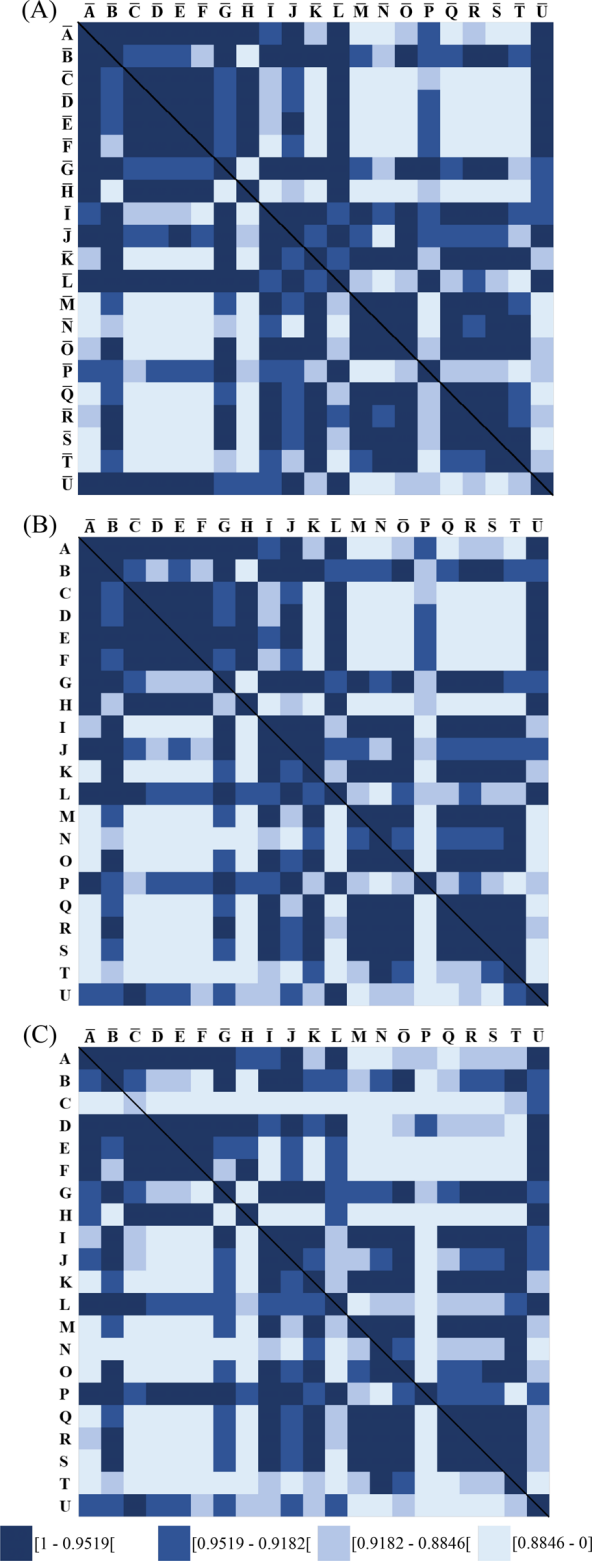
Spectral similarity score (SSS) barcode plots for similarity visualization. SSS were calculated between (A) averaged reference relative signal intensities, (B) an individual plate included in the reference group and the reference, and (C) an individual plate not included in the reference and the reference.

To evaluate the robustness of the SSS-barcodes, the SSS between an individual plate used for generating the reference (Figure 7B) or an individual plate not used for generating the reference (Figure 7C) and the reference were calculated. Similar barcodes were obtained, demonstrating the robustness of the SSS-barcode visualization, even when individual plates are compared to the reference. Additionally, the minimum SSS ((B) 0.6207, (C) 0.6595) and the average SSS ((B) 0.9120, (C) 0.9062) differed only slightly from the reference-to-reference SSS-barcode plot. One exception can be observed for emulsifier C, where SSS values were calculated between an individual plate not used for generating the reference and the reference data set (Figure 7C). This emulsifier showed low similarity with all references, even with its own reference. For this specific emulsifier, poor chromatography of the β signal group was observed. Instead of a single intense signal for β_2_, peak-broadening was detected, leading to an overall lower intensity of the signals. Thus, it is advisable to review the densitograms in cases of unexpected SSS.

SSS-barcoding appears suitable for the quality control of LACTEM emulsifiers, as the similarity between emulsifiers can be easily visualized in incoming goods inspection. Thus, nonconforming emulsifiers can be detected before they are added to the product, thereby avoiding recalls that result in economic losses. However, appropriate similarity thresholds for the application of LACTEM need to be defined for each food product, which requires an understanding of the influence of structural composition on techno-functional properties.

#### 3.3.4 Batch-to-Batch Variability of LACTEM Emulsifiers

Batch-to-batch variability of emulsifiers is highly important for ensuring consistent food quality. Thus, the capability of SSS-barcoding to detect batch-to-batch variability was explored. Comparing the SSS-barcode for two batches sampled within a two-year period, for four emulsifiers, three (N, O, and Q), showed similar barcodes, whereas one (P) showed notable variation in the SSS-barcode (Figure 8). This was even more distinct when differences of the SSS for batches of the same emulsifier were calculated (Figure S5). Nevertheless, looking at the specific SSS of emulsifier P in contrast to its reference, comparable results (0.99394 for P_1_ and 0.98270 for P_2_) were achieved, indicating high batch-to-batch consistency. The reasons for this observation remain unclear but may be related to subtle differences in nonmajor components that affect overall fingerprint similarity. This example illustrates the method’s suitability for batch comparison. To date, it remains to be clarified whether and how these changes in the SSS-barcode influence techno-functional properties.

**Figure 8:**
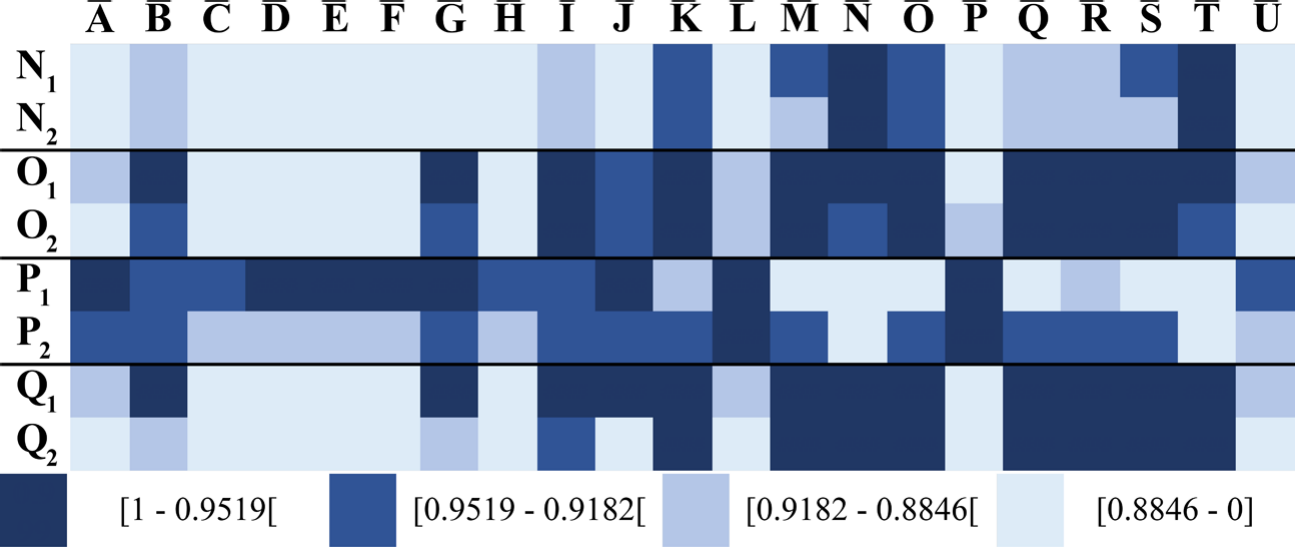
Spectral similarity score (SSS)-barcode plots for similarity visualization of different batches of LACTEM emulsifiers. Subscript indicates batch number.

#### 3.3.5 Application on LACTEM-Containing Products and Non-LACTEM Food Emulsifiers

Another question is whether LACTEM-containing products can be distinguished from other food emulsifiers, such as MG/DG, ACETEM, CITREM, and DATEM, based on SSS-barcoding. These emulsifiers share some of the common peaks, such as MG, DG, and TG. On the market, emulsifier products often contain LACTEM along with other ingredients. Skimmed milk powder, hydrolyzed starch syrup, glucose syrup, and milk protein are often added to these LACTEM-containing products. During the extraction of the LACTEM components from the emulsifier products, water-soluble ingredients are partitioned into the aqueous phase. However, ingredients such as milk proteins are surface-active^34^ and might influence the techno-functional properties of food products. Techno-functional properties cannot be expected to be reflected in a chromatographic similarity assessment and are therefore not captured by the current method. In addition, a fat component is often added to the emulsifier product. These fats comprise both hydrated and nonhydrated vegetable fats and oils, as well as animal fats like butterfat. Unlike nonlipid ingredients, these ingredients largely consist of acylglycerols, mainly TG, that are also part of the LACTEM emulsifier, and thus, are visualized with primuline.

Determination of the SSS of eight LACTEM-containing products (AA-AH) showed very low similarity (average SSS of 0.4210) of the samples to the reference, indicated by the light blue color (Figure 9). Inspection of the densitograms revealed that a high content of TG, reflected in an enlarged signal δ_1_, simultaneously led to lower signal intensities of the other signals, which was responsible for the low similarity (Figure S6). Thus, signal selection needs to be adjusted for the SSS analysis in fatty matrices. For process control, this aspect might be particularly important, as the composition of food emulsifiers can be detected at-line during food product manufacturing. There was one exception showing an SSS of up to 0.9753 for the LACTEM-containing product AA, indicating that the LACTEM content was higher in this LACTEM-containing product than in the others.

**Figure 9:**
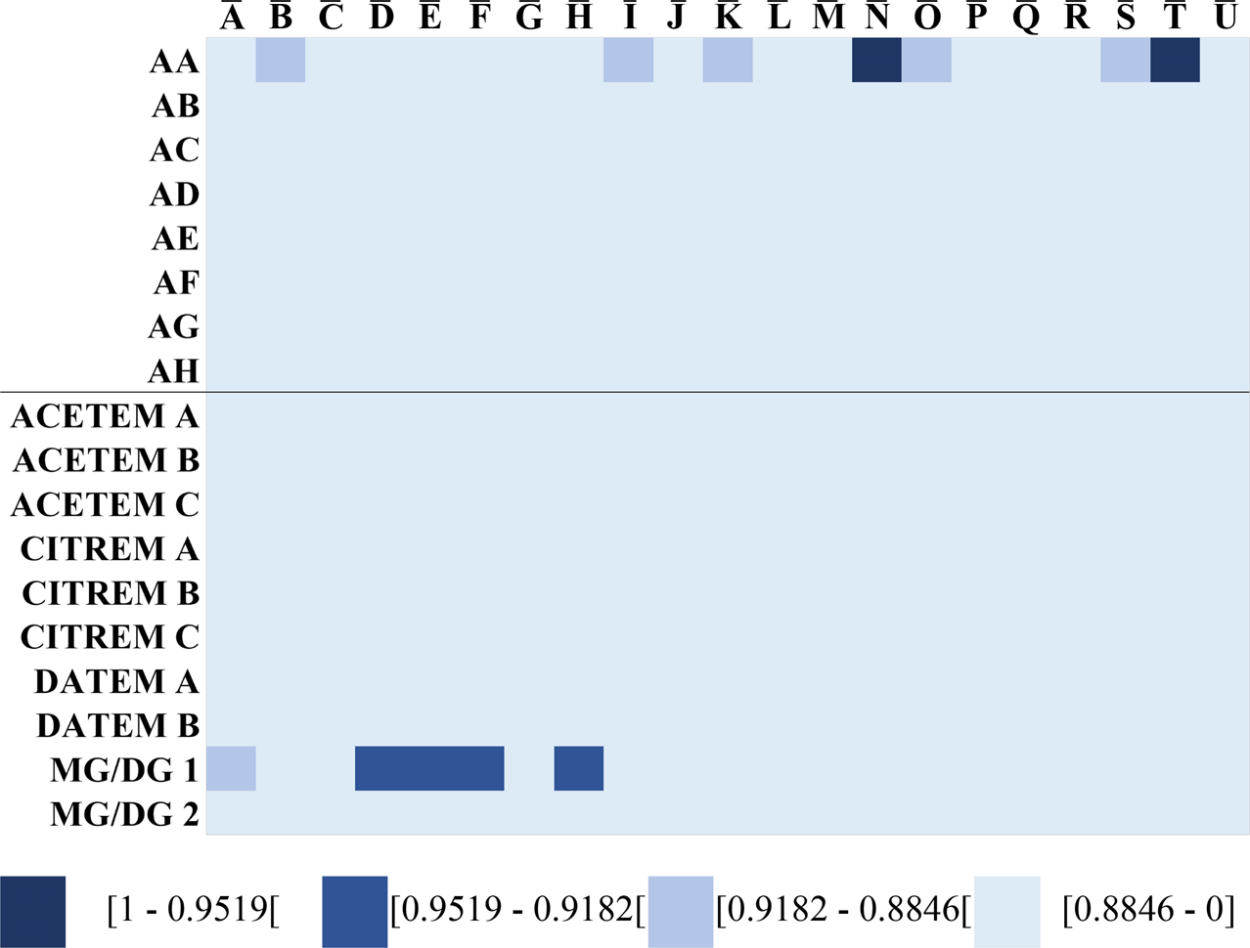
Spectral similarity score (SSS)-barcode plots for similarity visualization of LACTEM-containing products and non-LACTEM emulsifiers. SSS were calculated between the averaged reference relative signal intensities and LACTEM-containing products (AA-AI) and non-LACTEM emulsifiers (ACETEM, CITREM, DATEM, MG/DG).

By visual inspection of the densitograms, differentiation between pure LACTEM and non-LACTEM emulsifiers is possible. This was also reflected in the SSS-barcode plots, which showed low SSS with an average of 0.4223 (Figure 9) for the non-LACTEM emulsifiers with the LACTEM references. Sample MG/DG 1 deviated from this, showing SSS up to 0.9510 with reference H, indicating high structural similarity with LACTEM. This is explained by the production of LACTEM from MG/DG. LACTEM-containing products showed, with an average SSS of 0.4210, low similarity with LACTEM as well. These LACTEM-containing products and emulsifiers such as MG/DG, ACETEM, CITREM, or DATEM cannot be distinguished based on the present SSS-barcoding. Even extending the color-coding intervals in 0.3 steps did not allow differentiation between LACTEM-containing products and other non-LACTEM emulsifiers. This can be explained by the fact that the SSS were calculated exclusively based on *h*R_F_ values characteristic of LACTEM emulsifiers. Thus, LACTEM-containing products cannot be reliably analyzed using the developed SSS-barcode method, showing an important limitation of the current proposed method. To solve this issue, peak selection might have a significant influence. This involves omitting the TG but also adding different signals for different emulsifiers, thereby taking into account the different fingerprints. Furthermore, factors that influence techno-functionality, but are not considered in the proposed method, could be included.

### 3.4 Partial Least Squares Regression

The presented results showed that similarity between LACTEM emulsifiers varied up to 33% (SSS ranges between 0.6658 and 1). This raises the question of whether these dissimilarities influence the techno-functional properties of food products. To explore the relationship between chromatographic signal intensities and techno-functional properties in aerosol whipping cream, PLSR was applied in a proof-of-concept approach using four LACTEM emulsifiers and four LACTEM-containing products that showed variation in their SSS-barcodes. To account for the nonlinear fluorescence response of the densitometric data, the signal intensities of the common peaks were log10-transformed prior to modeling. This led to a reduced RMSEP and higher explained variance, thereby improving model performance. For each techno-functional property, the optimal number of components was determined based on minimum RMSEP (Figure S7). A comparison of the predicted results with the actual results is exemplarily shown for foam firmness (Figure 10). Sample AB was not foamable and was therefore assigned a foam firmness of 0 mN, but was still included in model calibration. Due to model extrapolation, the predicted value was slightly negative, which is not physically meaningful. As this was a proof-of-concept with a limited number of samples, the predictions should be interpreted with caution. The model does not reveal which specific components, or their concentration, are responsible for the prediction. It is conceivable that certain components need to exceed a threshold concentration to achieve a higher foam firmness, but further research with larger data sets and possibly fractionated emulsifiers would be required to investigate this as the current study was strictly of exploratory character. The PLSR models of further techno-functional properties (normalized drainage, apparent viscosity, D_90,3_, and overrun) showed similar trends (Figures S8–S11). The ratio of RMSEP_LOO_/RMSEP_Residues_ indicated slight overfitting for the normalized drainage, foam firmness, and D_90,3_ (RMSEP_LOO_/RMSEP_residues_ approximately 1.5), moderate overfitting for the overrun (RMSEP_LOO_/RMSEP_residues_ approximately 2.25), and strong overfitting for the viscosity (RMSEP_LOO_/RMSEP_residues_ approximately 3).

**Figure 10:**
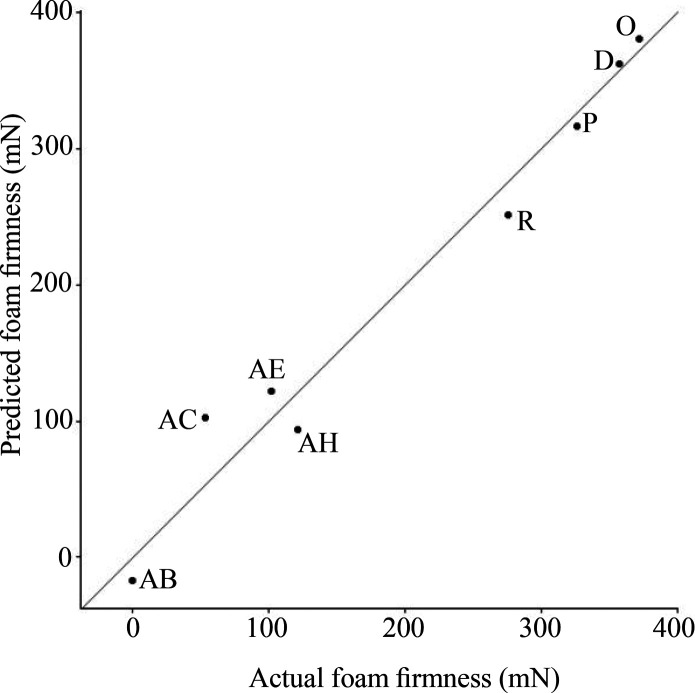
Comparing predicted and actual foam firmness (mN) of LACTEM emulsifiers (D, O, P, R) and LACTEM-containing products (AB, AC, AE, AH). PLSR was performed using two components based on leave-one-out-cross-validation. Sample AB was not foamable, and thus, foam firmness was not determined and set to 0 mN. The slightly negative predicted value results from model extrapolation and has no physical meaning.

Based on the available data, the prediction of techno-functional properties for LACTEM-containing products might be impaired compared to pure LACTEM emulsifiers. These products contain further surface-active ingredients, such as milk proteins. These are not considered in the proposed method, resulting in discrepancies between the chromatographic fingerprint and techno-functional properties. This shows a major limitation for the modeling of techno-functional properties. Moreover, the limited sample size prevents any proven predictive conclusions, and the PLSR analysis was strictly exploratory. Nevertheless, despite these limitations, the results demonstrate the potential of HPTLC as a basis for future predictive approaches, provided that larger data sets and independent validation are available.

## Supplementary Material



## Data Availability

All recent data are available from the corresponding author upon reasonable request.

## Notes

Information about the composition of the LACTEM-containing products, the variation of the *h*R_F_ values and signal intensity ratios, the mean relative peak intensities of the common peaks (normalized to the IS) for the LACTEM reference, the *U* in relation so relative signal intensity (normalized to the IS), the differences in the SSS across plates, the densitometric data of a LACTEM-containing product AC, RMSEP for component selection of the PLSR, and comparison of predicted and actual values for the normalized drainage, the overrun, D_90,3_, and apparent viscosity; Graphic for table of contents (PDF)

## Abbreviations Used:

ANOVA: analysis of variance
CV: coefficient of variation
DG: diacylglycerols
FA: fatty acids
FLD: fluorescence detection
GC: gas chromatography
HLB: hydrophilic–lipophilic balance
HPLC: high-performance liquid chromatography
HPTLC: high-performance thin-layer chromatography
IS: internal standard
LOO cross-validation: leave-one-out cross-validation
MG: monoacylglycerols
MS: mass spectrometry
U: half-width of the 95% confidence interval
PLSR: partial least squares regression
RMSEP: root mean squared error of prediction
SSS: spectral similarity scores
TG: triacylglycerols
UV: ultraviolet
