# Supplementary material for: Spectral Similarity Score (SSS)-Barcoding for the Quality Control of LACTEM Emulsifiers by High-Performance Thin-Layer Chromatography
Source: J Agric Food Chem. 2026 Feb 26;74(9):7789–99. doi: 10.1021/acs.jafc.5c11928 (PMC12983357; doi:10.1021/acs.jafc.5c11928)
Supplement: Supplementary file 1 [file jf5c11928_si_001.pdf]

**Supplementary Information**

# Spectral Similarity Score (SSS)-Barcoding for the Quality Control of LACTEM Emulsifiers by High-Performance Thin-Layer Chromatography

Katharina Schuster[1], Sedef Torun[1], Inès Kainz[2], Max Schwarz-Blankart[2], Jörg Hinrichs[2], Panagiotis Steliopoulos[3], Claudia Oellig[1,4]*

[1]Department of Food Chemistry and Analytical Chemistry (170a), Institute of Food Chemistry, University of Hohenheim, Garbenstrasse 28, 70599 Stuttgart, Germany

[2]Department of Soft Matter Science and Dairy Technology (150e), Institute of Food Science and Biotechnology, University of Hohenheim, Garbenstrasse 21, 70599 Stuttgart, Germany

[3]Chemisches und Veterinäruntersuchungsamt (CVUA), Weißenburgerstrasse. 3, 76187 Karlsruhe, Germany

[4]Justus Liebig University Giessen, Institute of Food Chemistry and Food Biotechnology, Heinrich-Buff-Ring 17-19, 35392 Giessen, Germany

*Corresponding author Tel.: +49-641-99-34960

E-mail address: claudia.oellig@uni-giessen.de

Table S1: Composition of the LACTEM-containing products, besides LACTEM.

| LACTEM-containing product | Ingredients |
|---|---|
| AA | hydrated palm fat, skimmed milk powder |
| AB | hydrolysed starch syrup, hydrated plant oils, hydrated coconut oil |
| AC | hydrolysed starch syrup, hydrated plant oils, hydrated coconut oil |
| AD | hydrolysed starch syrup, palm kernel oil |
| AE | animal fats, milk proteins, hydrolysed starch syrup, butter fat |
| AF | hydrolysed starch syrup, hydrated plant oils |
| AG | C8-C18 acylglycerols, C18 unsaturated acylglycerols, dehydrated grain syrups, palm oil |
| AH | coconut oil, glucose syrup |

Table S2: Coefficient of variation (*CV)* (%) for the *h*$R_F$ values of the signals $\alpha_1$-$\delta_1$ of LACTEM emulsifiers ($n$ = 4) for different parameter settings.

| Parameter | Develop-ment | *CV* (%) | | | | | | | |
|---|---|---|---|---|---|---|---|---|---|
| | | Signal $\alpha_1$ | Signal $\alpha_2$ | Signal $\alpha_3$ | Signal $\alpha_4$ | Signal $\beta_1$ | Signal $\beta_2$ | Signa l $\gamma_1$ | Signal $\delta_1$ |
| Without chamber climate control | 1+2 | 6.8 | 8.5 | 8.3 | 7.6 | 1.1 | 1.1 | 2.7 | 0.6 |
| Chamber saturation | 1 | 3.5 | 3.7 | 3.3 | 2.7 | 1.0 | 1.4 | 2.4 | 0.2 |
| | 2 | 5.4 | 5.4 | 4.8 | 4.0 | 1.2 | 1.1 | 2.0 | 0.5 |
| | 1 + 2 | 6.7 | 7.2 | 7.6 | 5.9 | 2.3 | 2.6 | 4.2 | 1.2 |
| Plate conditioning | 1 | 4.8 | 5.8 | 5.2 | 3.9 | 1.7 | 1.1 | 0.1 | 1.5 |
| | 2 | 4.4 | 4.6 | 4.4 | 3.6 | 0.1 | 0.2 | 0.4 | 0.3 |
| | 1 + 2 | 3.3 | 4.3 | 3.7 | 3.3 | 1.3 | 0.3 | 0.5 | 0.5 |
| Chamber saturation + plate conditioning | 1 | 3.8 | 2.7 | 2.2 | 2.2 | 0.7 | 0.8 | 1.7 | 0.8 |

Table S3: Intra-plate deviation expressed as coefficient of variation (*CV*) (%) of $hR_F$ values ($n$ = 21) of the signals $\alpha_1$-$\delta_1$.

| *CV* (%) | | | | | | | |
|---|---|---|---|---|---|---|---|
| Signal $\alpha_1$ | Signal $\alpha_2$ | Signal $\alpha_3$ | Signal $\alpha_4$ | Signal $\beta_1$ | Signal $\beta_2$ | Signal $\gamma_1$ | Signal $\delta_1$ |
| 1.3 | 1.4 | 1.3 | 1.2 | 0.6 | 0.5 | 0.6 | 0.4 |

Table S4: Stability of the boric-acid impregnation with regard to the $hR_F$ value over the range of a working week.

| Day | $hR_F$ |
|---|---|

| | Signal $\alpha_1$ | Signal $\alpha_2$ | Signal $\alpha_3$ | Signal $\alpha_4$ | Signal $\beta_1$ | Signal $\beta_2$ | Signal $\gamma_1$ | Signal $\delta_1$ |
|---|---|---|---|---|---|---|---|---|
| 0 | 33.9 | 37.9 | 42.4 | 48.2 | 61 | 63.5 | 67.6 | 90.0 |
| | 36.9 | 41.8 | 46.2 | 51.7 | 59.5 | 62.2 | 67.6 | 89.7 |
| | 36.1 | 40.5 | 45.5 | 51.7 | 60.2 | 62.7 | 77.1 | 82.5 |
| | 35.9 | 40.5 | 45.6 | 51.7 | 60.2 | 62.7 | 77.1 | 82.5 |
| 2 | 34.2 | 38.1 | 42.6 | 48.2 | 60.8 | 63.2 | 66.9 | 89.9 |
| | 39.0 | 43.3 | 47.9 | 53.2 | 61.0 | 63.2 | 68.9 | 89.6 |
| | 37.4 | 42.4 | 47.4 | 53.8 | 62.4 | 64.6 | 74.5 | 84.5 |
| | 37.0 | 42.2 | 47.3 | 53.7 | 62.4 | 64.5 | 77.0 | 83.8 |
| 4 | 37.2 | 41.9 | 46.8 | 51.7 | 59.9 | 62.4 | 67.8 | 89.7 |
| | 38.9 | 43.2 | 47.8 | 52.9 | 61.1 | 63.2 | 67.8 | 89.6 |
| | 39.7 | 44.8 | 49.4 | 54.4 | 61.6 | 63.9 | 78.2 | 86.0 |
| | 39.3 | 44.7 | 49.1 | 54.2 | 61.7 | 63.9 | 80.3 | 85.7 |

Table S5: Mean of the signal ratios for emulsifier reference (ref) A-U and relative half-width of the 95% confidence interval (*U*) (%) expressed as the half-width of the 95% confidence interval. The number of replicates for each emulsifier varied between 6 and 8. For better clarity, numbers are expressed with two significant figures.

| Emulsifier ref | | A | B | C | D | E | F | G | H | I | J | K | L | M | N | O | P | Q | R | S | T | U |
|---|---|---|---|---|---|---|---|---|---|---|---|---|---|---|---|---|---|---|---|---|---|---|
| $\alpha_1/\alpha_1$ | mean | 1.0 | 1.0 | 1.0 | 1.0 | 1.0 | 1.0 | 1.0 | 1.0 | 1.0 | 1.0 | 1.0 | 1.0 | 1.0 | 1.0 | 1.0 | 1.0 | 1.0 | 1.0 | 1.0 | 1.0 | 1.0 |
| | *U* (%) | - | - | - | - | - | - | - | - | - | - | - | - | - | - | - | - | - | - | - | - | - |
| $\alpha_1/\alpha_2$ | mean | 1.3 | 0.71 | 1.8 | 2.0 | 1.9 | 2.5 | 0.71 | 4.8 | 0.60 | 0.74 | 0.67 | 1.8 | 0.55 | 0.61 | 0.56 | 1.3 | 0.64 | 0.57 | 0.54 | 0.64 | 1.6 |
| | *U* (%) | 16 | 21 | 23 | 17 | 20 | 22 | 18 | 35 | 17 | 20 | 16 | 20 | 14 | 18 | 22 | 22 | 16 | 17 | 16 | 19 | 20 |
| $\alpha_1/\alpha_3$ | mean | 2.9 | 1.7 | 4.0 | 4.8 | 4.5 | 7.4 | 1.8 | 10.2 | 1.3 | 1.8 | 1.3 | 4.1 | 1.1 | 1.5 | 1.1 | 3.1 | 1.4 | 1.4 | 1.3 | 1.8 | 4.7 |
| | *U* (%) | 24 | 22 | 31 | 26 | 29 | 41 | 23 | 50 | 16 | 23 | 20 | 31 | 12 | 16 | 20 | 26 | 15 | 16 | 17 | 20 | 23 |
| $\alpha_1/\alpha_4$ | mean | 5.5 | 3.0 | 9.9 | 12 | 11 | 16 | 3.3 | 26 | 2.3 | 3.9 | 2.6 | 8.0 | 1.8 | 2.6 | 2.4 | 7.2 | 2.1 | 2.5 | 2.2 | 3.7 | 9.8 |
| | *U* (%) | 34 | 29 | 47 | 40 | 47 | 52 | 33 | 71 | 22 | 29 | 25 | 45 | 18 | 22 | 21 | 31 | 19 | 19 | 18 | 26 | 32 |
| $\alpha_1/\beta_1$ | mean | 2.0 | 1.3 | 3.2 | 2.9 | 2.6 | 2.9 | 1.1 | 3.4 | 0.78 | 1.3 | 0.73 | 1.7 | 0.54 | 0.93 | 0.66 | 0.73 | 0.54 | 0.55 | 0.58 | 1.2 | 2.6 |
| | *U* (%) | 20 | 27 | 16 | 13 | 16 | 17 | 34 | 14 | 30 | 22 | 20 | 28 | 28 | 27 | 12 | 33 | 22 | 17 | 26 | 28 | 36 |
| $\alpha_1/\beta_2$ | mean | 0.56 | 0.52 | 0.80 | 0.59 | 0.58 | 0.63 | 0.47 | 0.71 | 0.48 | 0.48 | 0.70 | 0.79 | 0.64 | 1.2 | 0.58 | 0.38 | 0.64 | 0.51 | 0.61 | 1.2 | 0.90 |
| | *U* (%) | 16 | 20 | 22 | 16 | 15 | 19 | 17 | 17 | 19 | 14 | 16 | 17 | 23 | 21 | 16 | 24 | 23 | 17 | 26 | 21 | 19 |
| $\alpha_1/\gamma_1$ | mean | 6.3 | 7.5 | 12 | 6.2 | 5.3 | 6.6 | 7.0 | 6.0 | 8.7 | 6.0 | 7.1 | 8.6 | 11 | 25 | 11 | 16 | 12 | 12 | 10 | 8.5 | 8.8 |
| | *U* (%) | 29 | 26 | 29 | 15 | 37 | 29 | 44 | 20 | 37 | 11 | 33 | 23 | 42 | 66 | 25 | 36 | 32 | 25 | 21 | 16 | 20 |
| $\alpha_1/\delta_1$ | mean | 3.0 | 2.9 | 15 | 4.4 | 4.2 | 2.9 | 1.7 | 5.8 | 2.2 | 2.3 | 2.9 | 4.0 | 2.4 | 11 | 2.6 | 1.3 | 2.3 | 2.7 | 1.7 | 13 | 19 |
| | *U* (%) | 16 | 24 | 30 | 19 | 21 | 16 | 20 | 27 | 26 | 17 | 25 | 18 | 26 | 22 | 15 | 22 | 24 | 22 | 26 | 15 | 6.9 |

Table S6: Mean relative intensities (normalized to the internal standard) for the signals $\alpha_1$-$\delta_1$ of the emulsifier references (ref) A-U ($n$ = 6-8). For better clarity, numbers are expressed with two significant figures.

| Emulsifier ref | Mean relative intensities | | | | | | | | | | | | | | | | | | | | |
|---|---|---|---|---|---|---|---|---|---|---|---|---|---|---|---|---|---|---|---|---|---|
| | A | B | C | D | E | F | G | H | I | J | K | L | M | N | O | P | Q | R | S | T | U |
| $\alpha_1$ | 2.7 | 1.9 | 3.4 | 3.1 | 2.4 | 2.8 | 1.5 | 3.1 | 1.7 | 2.0 | 1.6 | 2.6 | 1.3 | 1.7 | 1.7 | 1.8 | 1.5 | 1.6 | 1.4 | 1.9 | 3.1 |
| $\alpha_2$ | 2.1 | 2.6 | 1.9 | 1.5 | 1.3 | 1.1 | 2.1 | 0.67 | 2.9 | 2.7 | 2.3 | 1.4 | 2.4 | 2.8 | 2.9 | 1.4 | 2.3 | 2.9 | 2.6 | 3.0 | 2.0 |
| $\alpha_3$ | 0.90 | 1.1 | 0.81 | 0.64 | 0.55 | 0.41 | 0.79 | 0.36 | 1.3 | 1.1 | 1.2 | 0.63 | 1.1 | 1.1 | 1.5 | 0.57 | 1.0 | 1.2 | 1.0 | 1.1 | 0.65 |
| $\alpha_4$ | 0.50 | 0.61 | 0.35 | 0.29 | 0.26 | 0.22 | 0.46 | 0.21 | 0.75 | 0.52 | 0.61 | 0.35 | 0.69 | 0.65 | 0.68 | 0.26 | 0.68 | 0.64 | 0.59 | 0.51 | 0.32 |
| $\beta_1$ | 1.5 | 1.6 | 1.1 | 1.1 | 1.0 | 1.0 | 1.6 | 0.94 | 2.5 | 1.7 | 2.3 | 1.8 | 2.7 | 2.1 | 2.6 | 2.8 | 2.9 | 3.1 | 2.5 | 1.8 | 1.5 |
| $\beta_2$ | 4.8 | 3.5 | 4.1 | 5.2 | 4.2 | 4.5 | 3.2 | 4.4 | 3.6 | 4.2 | 2.3 | 3.3 | 2.2 | 1.5 | 2.8 | 4.8 | 2.4 | 3.2 | 2.3 | 1.7 | 3.5 |
| $\gamma_1$ | 0.45 | 0.25 | 0.3 | 0.50 | 0.75 | 0.49 | 0.23 | 0.53 | 0.21 | 0.33 | 0.23 | 0.30 | 0.12 | 0.078 | 0.15 | 0.12 | 0.13 | 0.14 | 0.14 | 0.22 | 0.36 |
| $\delta_1$ | 0.93 | 0.66 | 0.2 | 0.74 | 0.60 | 1.0 | 0.90 | 0.59 | 0.84 | 0.93 | 0.60 | 0.68 | 0.61 | 0.15 | 0.67 | 1.5 | 0.70 | 0.66 | 0.85 | 0.14 | 0.16 |

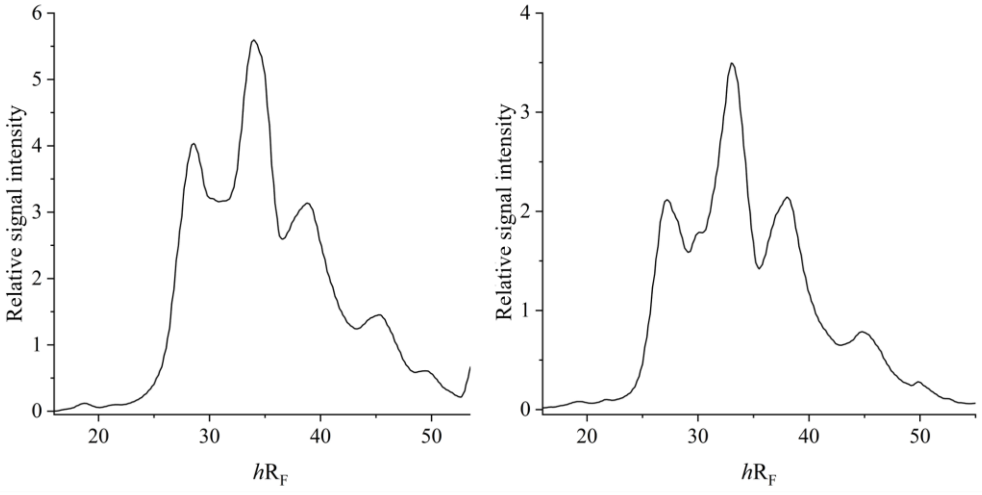


Figure S1: Cropped densitograms of LACTEM emulsifier D obtained at UV 366 nm (optical filter of K 400) in fluorescence mode, developed according to Schuster *et al.* (2023), showing the plate-to-plate variability of the signal of the α group.[11]

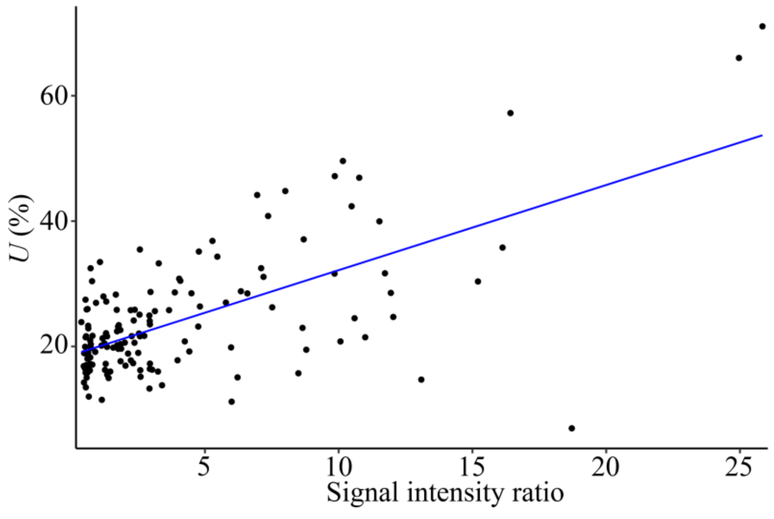


Figure S2: Scatter plot of the relative half-width of the 95% confidence interval ($U$ (%)) as a function of the relative signal intensity (normalized to the internal standard) ratio of signal $\alpha_1$ to a specific signal. The blue line represents the linear regression fit ($R^2$ = 0.41).

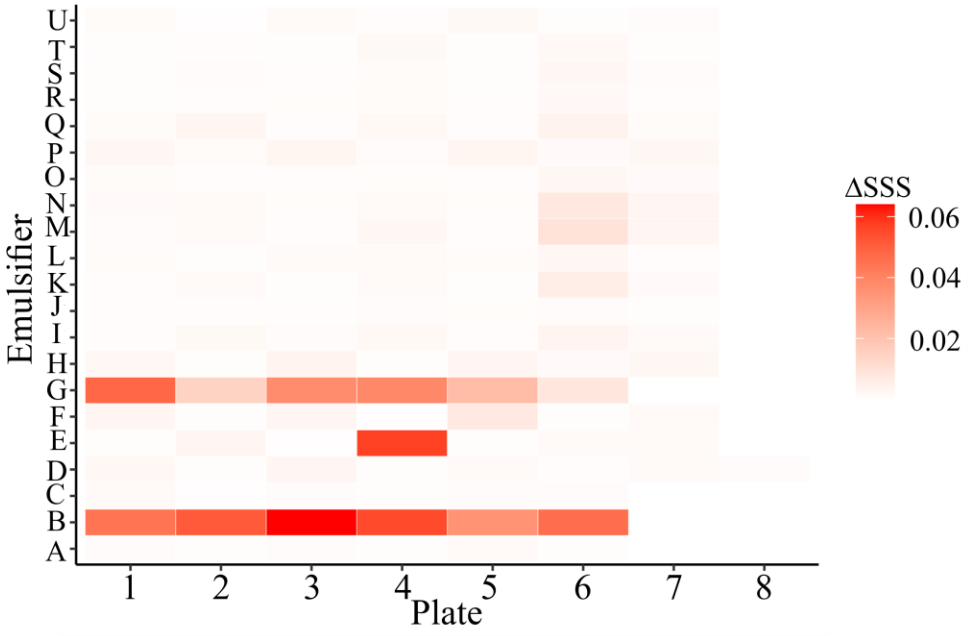


Figure S3: Heat map of the mean spectral similarity score differences (ΔSSS) for each emulsifier (A-U) and plate (6-8) combination. ΔSSS represents the difference between the SSS of the complete and the reduced peak (excluding $\alpha_4$ and $\gamma_1$) set.

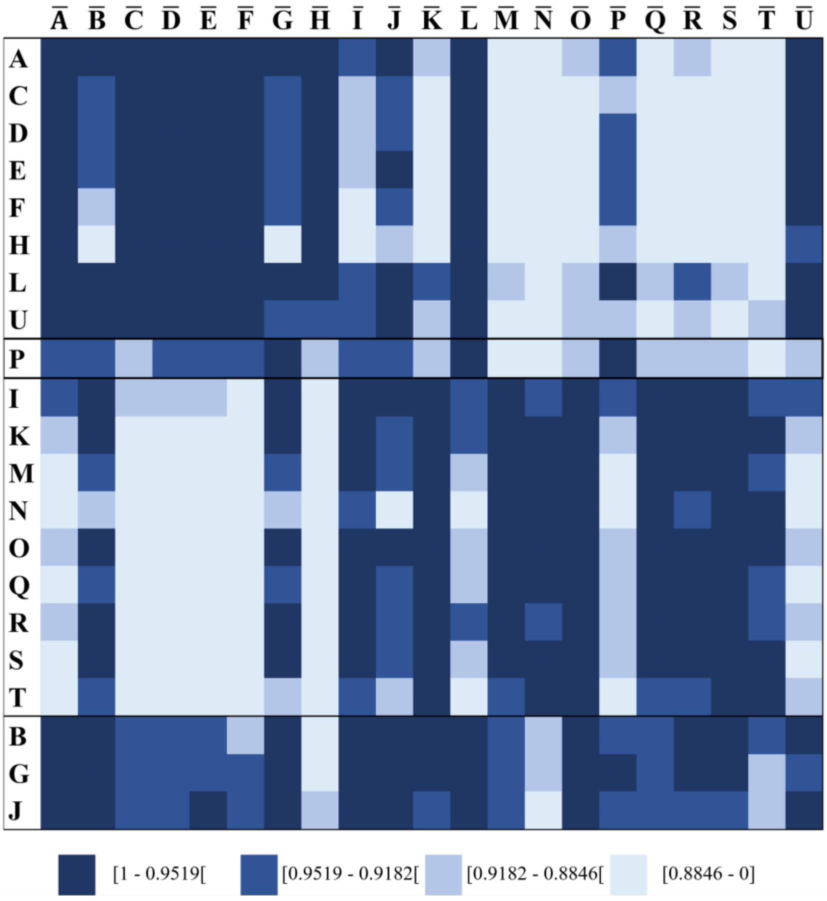


Figure S4: Sorted spectral similarity score (SSS) barcode plots for similarity visualization. SSS were calculated between averaged reference relative signal intensities.

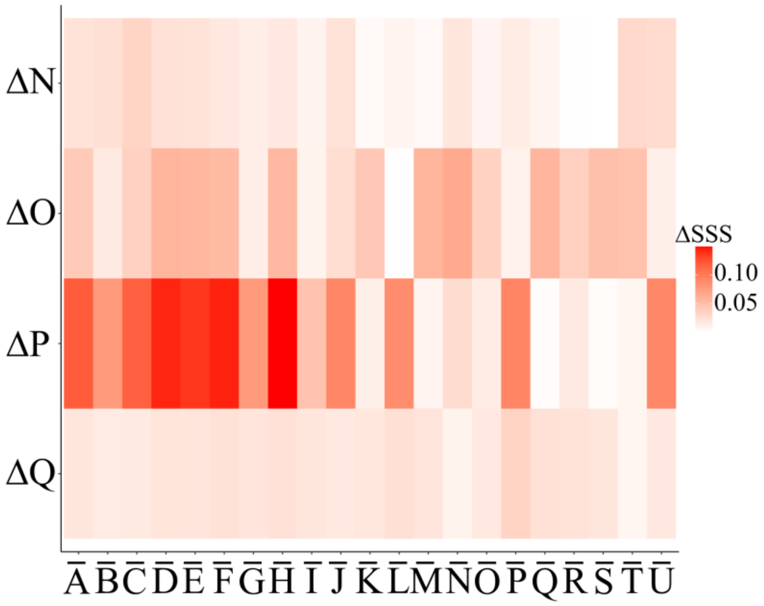


Figure S5: Heat map of the mean spectral similarity score differences (ΔSSS) for the two emulsifiers per batch. ΔSSS represents the difference between the first batch (N1, O1, P1, Q1) and the second batch (N2, O2, P2, Q2).

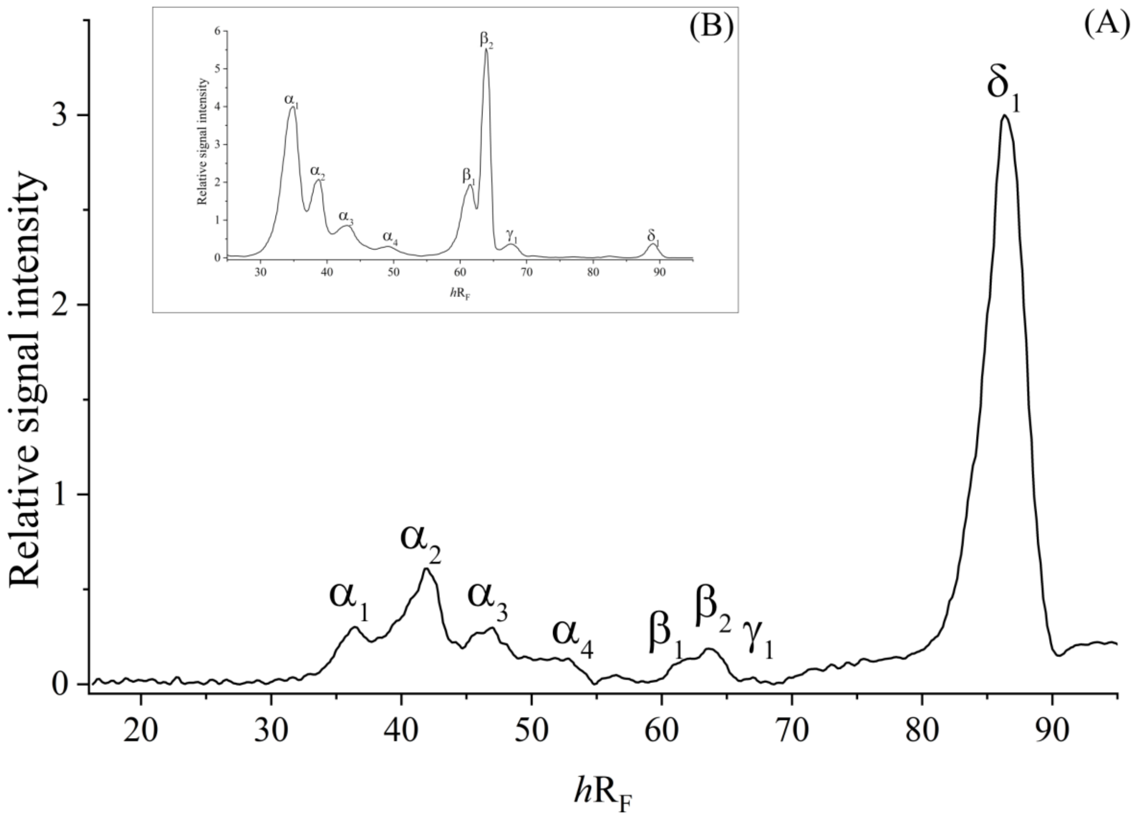


Figure S6: Densitogram of the LACTEM-containing product AC (A) and LACTEM emulsifier A (B) obtained at UV 366 nm (optical filter of K 400) in fluorescence mode, normalized to the internal standard.

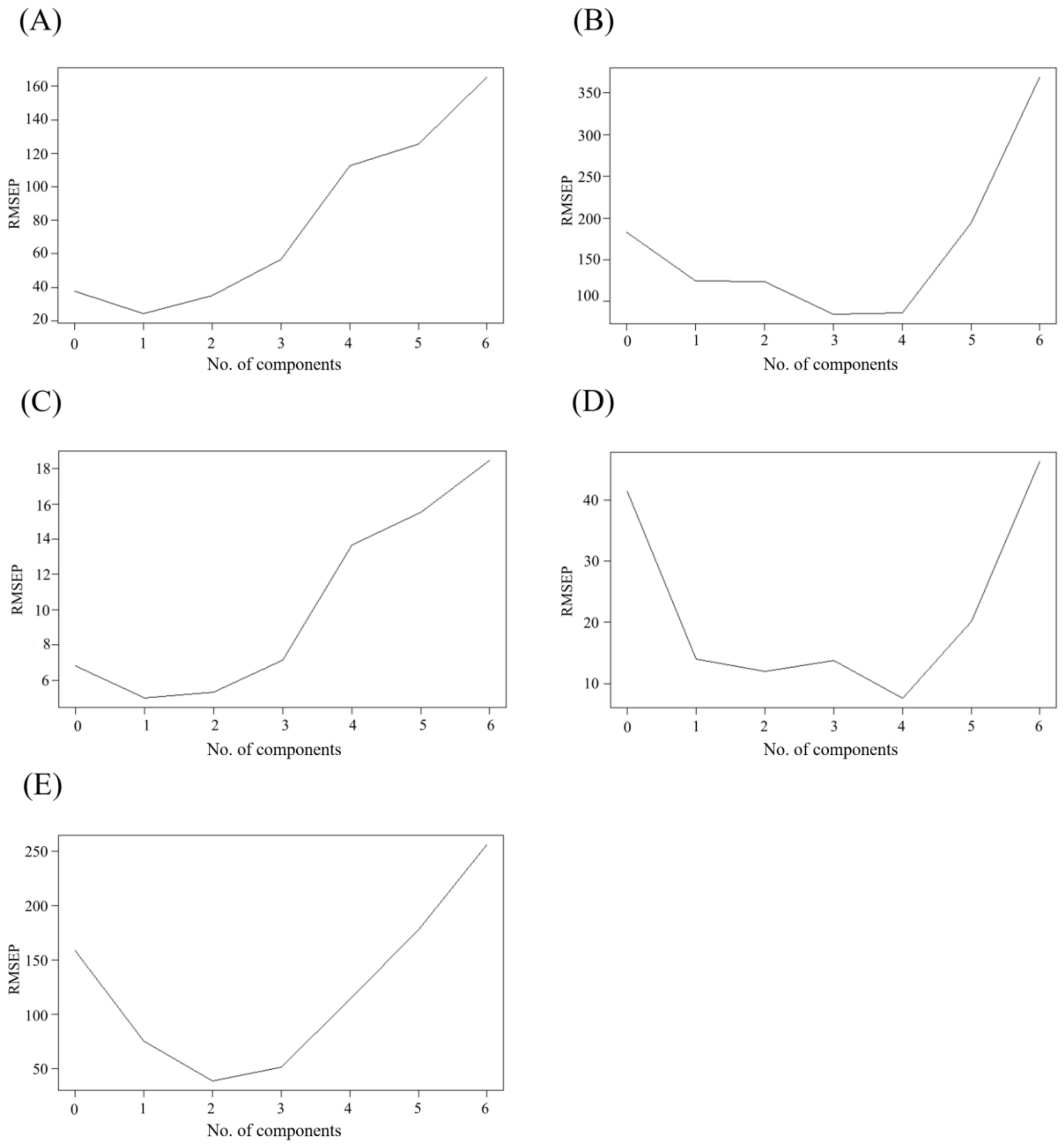


Figure S7: Root Mean Squared Error of Prediction (RMSEP) in relation to the No. of components for the PLSR models for (A) normalized drainage, (B) overrun, (C) $D_{90,3}$, (D) apparent viscosity, and (E) foam firmness.

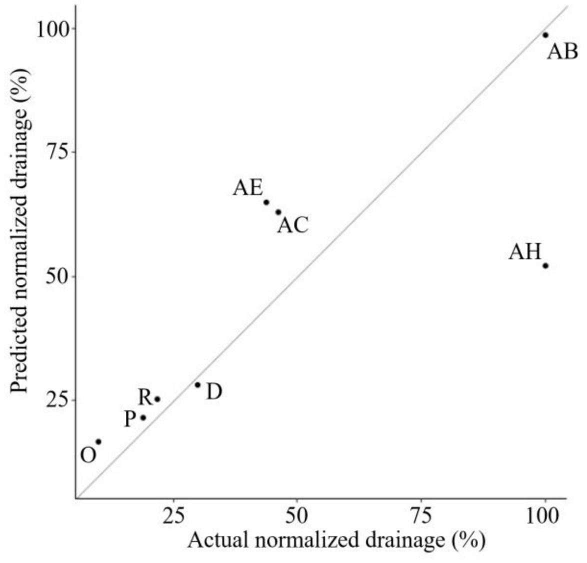


Figure S8: Comparing predicted and actual normalized drainage (%) of LACTEM emulsifiers (D, O, P, R) and LACTEM-containing products (AB, AC, AE, AH). PLSR was performed using one component based on leave-one-out-cross-validation.

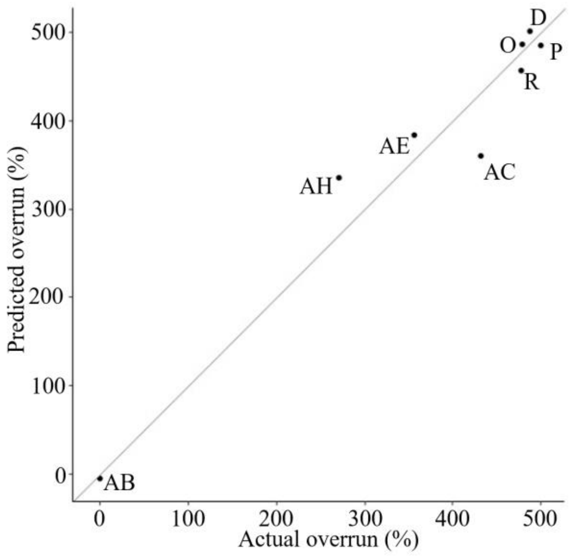


Figure S9: Comparing predicted and actual overrun (%) of LACTEM emulsifiers (D, O, P, R) and LACTEM-containing products (AB, AC, AE, AH). PLSR was performed using three components based on leave-one-out-cross-validation.

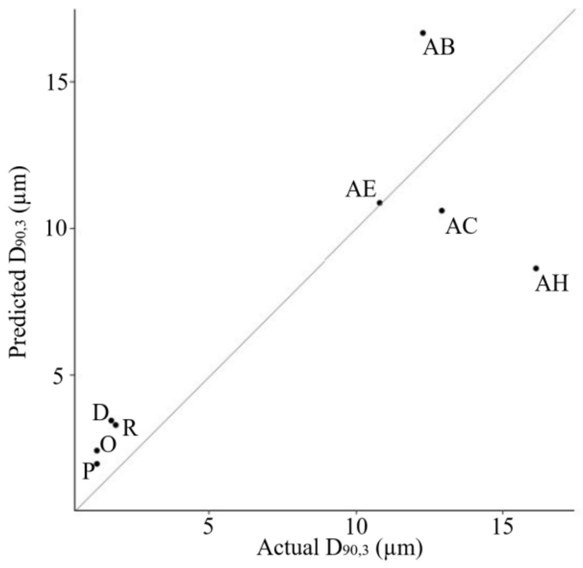


Figure S10: Comparing predicted and actual $D_{90,3}$ (μm) of LACTEM emulsifiers (D, O, P, R) and LACTEM-containing products (AB, AC, AE, AH). PLSR was performed using one component based on leave-one-out-cross-validation.

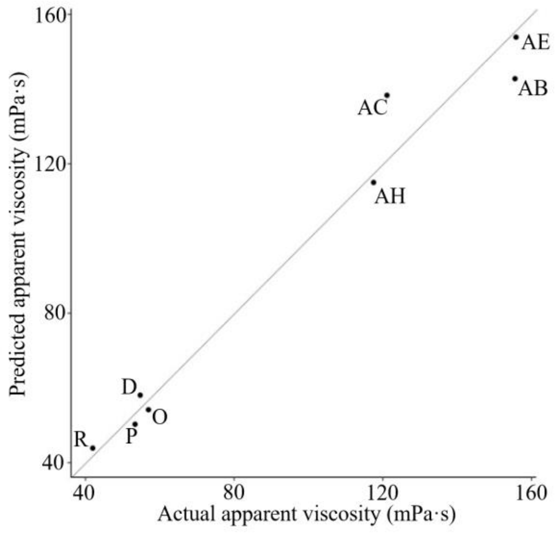


Figure S11: Comparing predicted and actual apparent viscosity (mPa·s) of LACTEM emulsifiers (D, O, P, R) and LACTEM-containing products (AB, AC, AE, AH). PLSR was performed using three components based on leave-one-out-cross-validation.
